# Hyaluronidase following buccal infiltrations of articaine with epinephrine for anesthesia of mandibular first molars: A split-mouth, double-blind, placebo-controlled randomized clinical trial

**DOI:** 10.4317/jced.59809

**Published:** 2022-11-01

**Authors:** Thiago-Pallin Gomes, Luiz-Felipe Palma, Maurício-José Tornelli, Helena-Regina Tornelli, Cíntia-Yuki Fukuoka, Maria-Aparecida Borsatti

**Affiliations:** 1 MSc. Graduate Dentistry Program, Ibirapuera University. São Paulo, SP, Brazil; 2 PhD. Graduate Dentistry Program, Ibirapuera University. São Paulo, SP, Brazil; 3 PhD. Faculty of Dentistry, Faculdades Metropolitanas Unidas. São Paulo, SP, Brazil; 4 PhD. Department of Biomaterials, Faculty of Dentistry, University of São Paulo. São Paulo, SP, Brazil; 5 PhD. Department of Stomatology, Faculty of Dentistry, University of São Paulo. São Paulo, SP, Brazil

## Abstract

**Background:**

Adjunctive hyaluronidase has been widely used for ophthalmic anesthesia; however, in Dentistry, very few studies are available so far. Thus, the present study aimed to evaluate anesthetic outcomes of adjunctive hyaluronidase administration following buccal infiltration of articaine with epinephrine for anesthesia of mandibular first molars.

**Material and Methods:**

Twenty-eight patients received a buccal supraperiosteal infiltration of 4% articaine with 1:100,000 epinephrine for anesthesia of the mandibular first molars, in a split-mouth approach. Afterward, randomly and using the same technique, they received either 1.0 mL of hyaluronidase (150 UTR/mL) or a placebo solution. Considering patients’ pain perceptions provoked by electric and mechanical stimulations, as well as using a pain scale, success rate, action onset time, duration of both pulpal and soft tissue anesthesia, and pain immediately after both punctures and on the 2nd day were assessed.

**Results:**

The pulpal anesthetic success rate was 85.7% for hyaluronidase and placebo groups. Soft tissue anesthesia showed a shorter action onset time and a longer duration when hyaluronidase was used; however, there was no difference between the groups regarding action onset time and duration of pulpal anesthesia. Pain at the puncture sites did not differ between the groups, regardless of the time point evaluated.

**Conclusions:**

Adjunctive hyaluronidase following buccal infiltration of articaine with epinephrine for mandibular first molars seems not to provide any advantage in anesthetic outcomes in which the nerve fibers are intraosseous (i.e., pulpal anesthesia). On the other hand, soft tissue anesthesia may be improved substantially by using this pharmacological strategy.

** Key words:**Hyaluronidase, local anesthesia, dentistry.

## Introduction

Hyaluronidase is an enzyme that exhibits exceptional tissue penetration, and is used as an adjunct to improve the absorption and dispersion of other injected drugs. Its mechanism is based on reversible hydrolysis of the β-1,4-glycosidic linkages of hyaluronic acid, a constituent of the extracellular matrix composed primarily of proteoglycans and glycoproteins synthesized by fibroblasts and their derived cells (1,2).

Before modern eye surgery techniques were developed, the associations of local anesthetic agents with bovine hyaluronidase had been commonly used in ophthalmic surgery to achieve less akinesia, leading to greater comfort and better patient and surgical satisfaction scores (3,4). The rationale for it was based on the premise that hyaluronidase would aid the spread of local anesthetic agents through the soft tissues around the eyes (5,6). Although several studies have investigated adjunctive hyaluronidase either when mixed in the local anesthetic solution (7-12) or with delayed administration (i.e., before the end of the anesthetic effect) (13,14) in many other organs and tissues, its possible benefits are still debated.

As traditional techniques for mandibular anesthesia are occasionally confounded by anatomical variations (15) and important clinical complications may arise during or after the procedure (e.g., intravascular injections, needle breakage, hematoma, trismus, and facial palsy) (16), alternative methods and substances may be of great value in Dentistry. Adjunctive hyaluronidase may facilitate the diffusion of local anesthetic agents (15), which may benefit medically compromised patients in whom sympathomimetic vasoconstrictors or long-acting local anesthetic agents should be avoided (17).

Also regarding the adjunctive use of hyaluronidase in Dentistry, the few studies conducted so far have yielded conflicting results. Whereas some authors have reported no benefit when hyaluronidase is injected during inferior alveolar nerve block (18), others have argued in favor of the use of this enzyme concomitantly to buccal infiltrations for mandibular teeth (19), as well as in combination with benzocaine gel applied over mandibular teeth presenting exposed pulp after the failure of intrapulpal anesthesia injection (5) or when injected late during inferior alveolar nerve block (13,14). Furthermore, reports of hypersensitivity to hyaluronidase following ophthalmic anesthesia have been recently published (20-22).

Taking into consideration the lack of studies on the late use of hyaluronidase in infiltrative terminal mandibular anesthesia, the present study aimed to evaluate the anesthetic outcomes of adjunctive hyaluronidase following buccal infiltration of articaine with epinephrine for mandibular first molars.

## Material and Methods

This split-mouth, double-blind, placebo-controlled randomized clinical trial was conducted at the Dental Clinic of the Faculty of Dentistry of the University of São Paulo (FOUSP) with a convenience sample, from February to November 2011. Sixty healthy outpatients, 18 years of age or older, who would receive resin composite restorations for two contralateral mandibular first molars (i.e., superficial enamel or shallow dentin caries on occlusal surfaces, with both teeth showing similar clinical and radiographic features) were selected.

Exclusion criteria consisted of systolic or diastolic arterial blood pressure >130 and >90 mmHg, respectively; heart rate <50 or >110 beats per minute; smoking; current pregnancy or lactation; systemic diseases or medications known to affect collagen or pain pathophysiology/perception; bone disorders; allergy or hypersensitivity to any drug used in the study protocol; previous radiotherapy of the head and neck; and local infection or inflammation.

All procedures were double-blinded and performed by a single researcher. Randomization was performed using a computer-generated Table, and both codes for each patient (right/left sides and experimental or placebo solutions) were then allocated into numbered, opaque, sealed envelopes by another researcher. According to the split-mouth study design, each patient attended clinical appointments twice (at least 7 days apart) and always in the same morning period.

Following a blood aspiration test to avoid accidental intravascular injection, the patients received a slow buccal supraperiosteal infiltration of 1.7 mL of 4% articaine hydrochloride with 1:100,000 epinephrine (Septanest with adrenaline 1:100,000™, Septodont, France) for anesthesia of a mandibular first molar. Shortly thereafter, either 1.0 mL of hyaluronidase (150 UTR/mL) or a placebo solution (distilled water), which were previously handled and placed into cartridges coded as A and B by the manufacturer, was injected at the same puncture area of the first injection and using the same buccal technique.

The success and duration of pulpal anesthesia were assessed according to the pain perception provoked by an electrical pulp stimulator (Vitality Scanner Pulp Vitality Tester™, SybronEndo, USA) applied to the buccal surface of the tooth. Considering that the voltage of this device corresponds to values on a 0-80 scale and that vital lower molar teeth present a sensibility response in the range of 30-70 (23), the baseline value was measured before anesthesia. The electrical stimuli were also applied every 2 minutes after the anesthetic injection until reaching a value of 80. Pulpal anesthesia was considered successful when the patient reported the absence of pain on two consecutive stimulations. Action onset time was calculated from the final moment of the anesthetic solution infiltration to the total loss of pain sensation. Duration of action was determined by considering the period between the total loss of painful sensation to pulp stimulation and partial recovery (i.e., any value lesser than 80 on the device display) evaluated every 5 minutes.

In contrast, buccal soft tissue anesthesia was assessed using nociceptive mechanical stimulation by introducing a dental explorer into the buccal mucosa until periosteal contact near the target lower molar tooth every 2 minutes. Soft tissue anesthesia and the action onset time were both considered achieved when there was no pain response to the mechanical stimulus. After reaching successful anesthesia, the above-mentioned procedure was repeated every 10 minutes to ascertain the duration of action, considering the period between the total loss of painful sensation and its partial recovery (pain reported to any degree).

A pain scale presenting values from 0 (no pain) to 5 (the worst pain imaginable) and written instructions on its use were provided to the patients at the first appointment. The patients were asked about pain from the anesthetic infiltration site immediately after the first (solution of articaine with epinephrine) and second (hyaluronidase or placebo) injections and asked again on the 2nd day by phone call.

This study was previously approved by the local Research Ethics Committee, School of Dentistry of the University of São Paulo (FOUSP) (Protocol #63/2009, FR 255186). The patients received information regarding the study before being submitted to any procedure, and those who agreed to participate read and signed the informed consent form.

Data were analyzed both descriptively and inferentially in the BioEstat 5.0™ software (Instituto Mamirauá, BRA). The differences between hyaluronidase and placebo groups were analyzed by the paired samples T-test for continuous data and by the Wilcoxon test for discrete ordinal data. Moreover, effect sizes were determined using G*Power 3.197™ software (Universität Kiel, Germany). The observed power for each parameter was calculated also by the same software and adopting α = 0.05, as follows: soft tissue latency, 91.6%; soft tissue duration, 72.7%; pulpal latency, 14.7%; pulpal duration, 20.8%; pulpal anesthetic success, undetermined; pain after the first puncture, 5.2%; pain after the second puncture, undetermined; pain on the second day, 6.7%.

## Results

**Figure 1:**
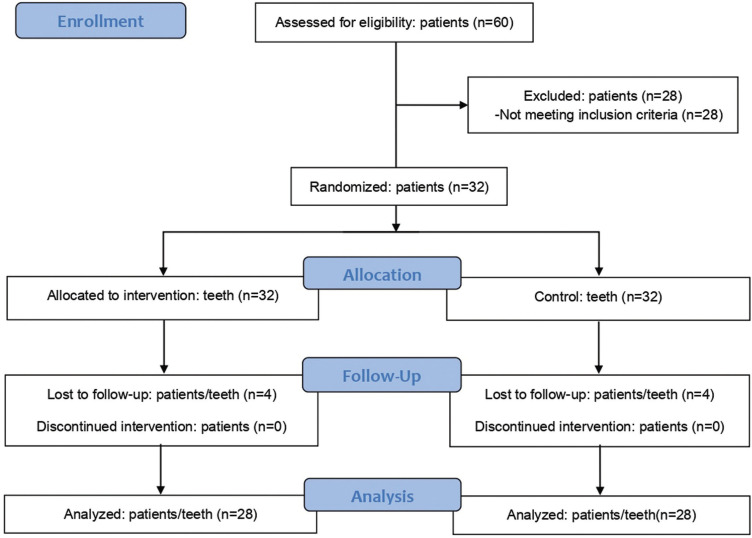
Flow diagram for patient inclusion and exclusion.

The final sample included 28 patients since 28 were initially excluded from the study and 4 did not complete all the proposed clinical procedures (Fig. 1). Twelve (43%) were men and 16 (57%) were women, with a mean age of 27.53 years (±6.56). The pulpal anesthetic success rate was 85.7% (24 patients) for hyaluronidase and placebo groups (*P* undetermined). A statistically significant shorter action onset time (*P* = 0.0018) and a longer duration (*P* = 0.0130) were observed in soft tissue anesthesia when hyaluronidase was used (Table 1). In contrast, no statistically significant intergroup differences were observed regarding either action onset time (*P* = 0.3584) or duration (*P* = 0.2467) of pulpal anesthesia (Table 2). Pain at the puncture sites was similar between the groups both immediately after the first puncture (*P* = 0.1698) and on the 2nd day (*P* = 1.0). In all cases, the patients reported the same score immediately after both second punctures (*P* undetermined) (Table 3).

**Table 1:**
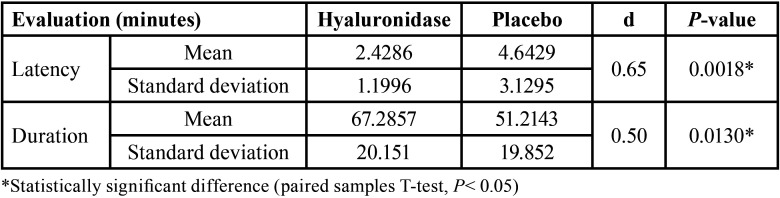
Average, standard deviation, and effect size (d) of evaluations of soft tissue anesthesia.

**Table 2:**
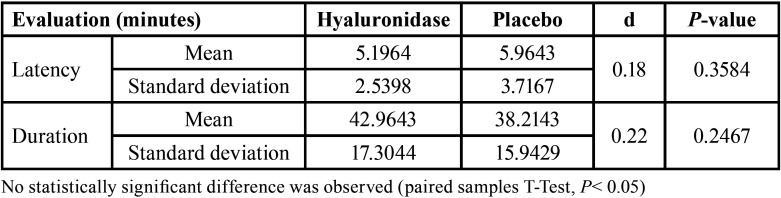
Average, standard deviation, and effect size (d) of evaluations of pulpal anesthesia.

**Table 3:**
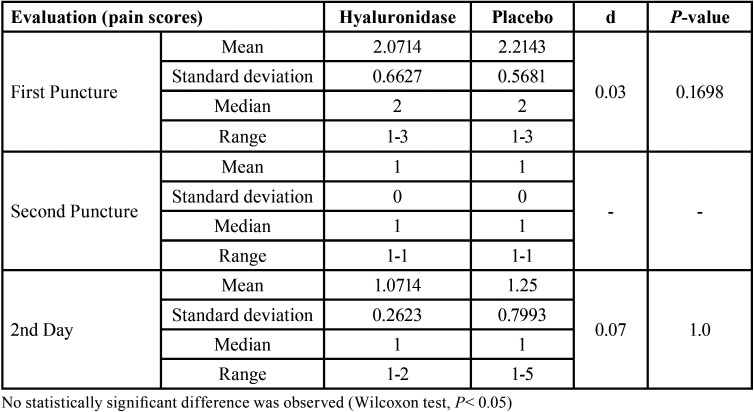
Average, standard deviation, median, range, and effect size (d) from evaluations of pain at the puncture sites.

## Discussion

This study evaluated the success rate, action onset time, and duration of both pulpal and soft tissue anesthesia and pain at puncture sites when hyaluronidase was used following buccal infiltration of articaine with epinephrine for mandibular first molars. To the best of the authors’ knowledge, this is the first paper addressing the adjunctive hyaluronidase with articaine and epinephrine for buccal mandibular anesthesia, as well as multiple anesthetic outcomes.

Buccal infiltrations with articaine are more effective than lidocaine ones for posterior mandibular teeth (24,25). Its improved efficacy is related to enhanced bone penetration due to greater lipid solubility that is conferred by a thiophene ring, an additional ester group (26), and intramolecular hydrogen bond formation (27).

The results of this study indicate that adjunctive hyaluronidase significantly prolonged the duration of soft tissue anesthesia, and also shortened its action onset time; however, no additional benefit to pulpal anesthesia was observed. The osseous extracellular matrix is calcified due to the deposition of hydroxyapatite crystals (comprised of calcium phosphate released by the rupture of vesicles in the maturation phase) on collagen fibrils and by condensation of existing components (hyaluronan) during osteogenesis (28,29). This condensation of the matrix probably hinders hyaluronidase action in mature bone (30), which may explain the present findings. Summing up, adjunctive hyaluronidase in the concentration proposed herein following buccal infiltrations may be an interesting strategy for procedures encompassing soft tissues in which long-lasting anesthesia with a fast action onset is desired such as periodontal surgeries and complex biopsies.

A certain degree of discomfort at the puncture sites was expected among patients in the hyaluronidase group, because of catabolism of connective tissue components that may remain incompletely restored for up to 48 hours (18,31). Curiously, there were no intergroup differences in pain at the injection sites either immediately after punctures or on the 2nd day, showing that the concentration of hyaluronidase used herein was suiTable for the buccal technique and does not seem to spread to neighboring tissues, since there were no reports of adverse effects such as trismus and pain as observed in the conventional alveolar inferior nerve block (18).

Because few studies on the use of hyaluronidase in dentistry have been completed, the present results are encouraging and can be considered a relevant contribution to the literature; however, these findings should be interpreted with caution. Firstly, the sample size was not determined statistically before patient enrollment, which may have yielded biased results, especially on the likelihood of failing to detect true differences between groups (type-2 error). Because statistical power and P-values depend simultaneously on sample and effect sizes, the convenience sample used herein could not provide sufficient power to detect some significant differences between both groups (32), as noted by the observed (posthoc) power calculated for each anesthetic outcome (when possible). Likewise, other variables need to be better investigated in order to obtain more robust data. These variables may include the concentrations of both hyaluronidase and vasoconstrictor, the use of other anesthetic agents, the selection of different mandibular/maxillary anatomic sites (i.e., where variable cortical bone thickness may affect the bioavailability of local anesthetics and hyaluronidase), the application for a limited and restricted purpose (aiming at achieving pulpal or soft tissue anesthesia), and the potential of preventing further bleeding.

Summing up, within the limitations of this study, adjunctive hyaluronidase following buccal infiltration of articaine with epinephrine for mandibular first molars seems not to provide any advantage in anesthetic outcomes in which the nerve fibers are intraosseous (i.e., pulpal anesthesia). On the other hand, soft tissue anesthesia may be improved substantially by using this pharmacological strategy.

## References

[1] Watson D (1993). Hyaluronidase. Br J Anaesth.

[2] Hynes WL, Walton SL (2000). Hyaluronidases of Gram-positive bacteria. FEMS Microbiol Lett.

[3] Rüschen H, Adams L, Bunce C (2013). Use of hyaluronidase as an adjunct to local anaesthetic eye blocks. Cochrane Database Syst Rev.

[4] Shea K, Stannard D (2021). Use of Hyaluronidase as an Adjunct to Local Anaesthetic Eye Blocks to Reduce Intraoperative Pain in Adults. AORN J.

[5] Sooraparaju SG, Abarajithan M, Sathish ES, Suryakumari NBP, Ealla KKR, Gade W (2015). Anaesthetic efficacy of topical benzocaine gel combined with hyaluronidase for supplemental intrapulpal injection in teeth with irreversible pulpitis- a double blinded clinical trial. J Clin Diagnostic Res.

[6] Buhren BA, Schrumpf H, Hoff N P, Bölke E, Hilton S, Gerber PA (2016). Hyaluronidase: from clinical applications to molecular and cellular mechanisms. Eur J Med Res.

[7] Eckenhoff JB, Kirby CK (1951). The use of hyaluronidase in regional nerve blocks. Anesthesiology.

[8] Kirby CK, Eckenhoff JE, Looby JP (1950). The use of hyaluronidase with local anesthetic agents in surgery and dentistry. Ann N Y Acad Sci.

[9] Mohamed AA, Radwan TA, Mohamed MM, Mohamed HA, Mohamed Elemady MF, Osman SH (2018). Safety and efficacy of addition of hyaluronidase to a mixture of lidocaine and bupivacaine in scalp nerves block in elective craniotomy operations; comparative study. BMC Anesthesiol.

[10] Gray TR, Dzikiti BT, Zeiler GE (2019). Effects of hyaluronidase on ropivacaine or bupivacaine regional anaesthesia of the canine pelvic limb. Vet Anaesth Analg.

[11] Koh WU, Min HG, Park HS, Karm MH, Lee KK, Yang HS (2015). Use of hyaluronidase as an adjuvant to ropivacaine to reduce axillary brachial plexus block onset time: a prospective, randomised controlled study. Anaesthesia.

[12] Tempestini-Horliana ACR, Lamers ML, Yonamine M, Aulestia-Viera PV, Santos MF Dos, Borsatti MA (2019). Late hyaluronidase injection in local anesthesia: Morphofunctional evaluation in rat sciatic nerve block. Indian J Dent Res.

[13] Tempestini-Horliana ACR, de Brito MAD, Perez FEG, Simonetti MPB, Rocha RG, Borsatti MA (2008). Hyaluronidase increases the duration of mepivacaine in inferior alveolar nerve blocks. J Oral Maxillofac Surg.

[14] Satish SV, Shetty KP, Kilaru K, Bhargavi P, Reddy ES, Bellutgi A (2013). Comparative evaluation of the efficacy of 2% lidocaine containing 1:200,000 epinephrine with and without hyaluronidase (75 IU) in patients with irreversible pulpitis. J Endod.

[15] Looby JP, Kirby CK (1949). Use of hyaluronidase with local anesthetic agents in dentistry. J Am Dent Assoc.

[16] Gillespie M, Gunsolly C (2020). Intracranial air embolism after inferior alveolar nerve block: a case report. Clin Pract Cases Emerg Med.

[17] Tornelli MJ, Prado RMS, Tornelli HR, Prado GF, Vieira PVA, Rocha RG (2016). Cardiovascular effects of combined hyaluronidase and mepivacaine in dental anesthesia: A randomized clinical trial. Am J Dent.

[18] Ridenour S, Reader A, Beck M, Weaver J (2001). Anesthetic efficacy of a combination of hyaluronidase and lidocaine with epinephrine in inferior alveolar nerve blocks. Anesth Prog.

[19] Etzler FE (1958). A clinical appraisal of hyaluronidase in dental procedures. Oral Surgery Oral Med Oral Pathol.

[20] Delaere L, Zeyen T, Foets B, Van Calster J, Stalmans I (2009). Allergic reaction to hyaluronidase after retrobulbar anaesthesia: a case series and review. Int Ophthalmol.

[21] Rajalakshmi A, Kumar Ma (2016). Hyaluronidase hypersensitivity: A rare complication of peribulbar block. Indian J Ophthalmol.

[22] Katahanas G, Van Nieuwenhuysen C, Park J, McKelvie J, McLintock C (2021). Hypersensitivity reaction to hyaluronidase following peribulbar anesthesia: a case series. Can J Ophthalmol.

[23] Barczak K, Palczewska-Komsa M, Wilk A, Nowicka A, Buczkowska-Radlińska J, Wiszniewska B (2020). Pulp sensibility to electric stimuli in the Caucasian population. Aust Endod J.

[24] Kanaa MD, Whitworth JM, Corbett IP, Meechan JG (2006). Articaine and lidocaine mandibular buccal infiltration anesthesia: a prospective randomized double-blind cross-over study. J Endod.

[25] Robertson D, Nusstein J, Reader A, Beck M, McCartney M (2007). The anesthetic efficacy of articaine in buccal infiltration of mandibular posterior teeth. J Am Dent Assoc.

[26] Ghadimi S, Shahrabi M, Khosravi Z, Behroozi R (2018). The efficacy of articaine infiltration versus lidocaine inferior alveolar nerve block for pulpotomy in mandibular primary second molars: randomized clinical trial. J Dent Res Dent Clin Dent Prospects.

[27] Skjevik ÅA, Haug BE, Lygre H, Teigen K (2011). Intramolecular hydrogen bonding in articaine can be related to superior bone tissue penetration: A molecular dynamics study. Biophys Chem.

[28] Setiawati R, Rahardjo P (2019). Bone Development and Growth. In: Yang H, editor. Osteogenes. Bone Regen.

[29] Zhai P, Peng X, Li B, Liu Y, Sun H, Li X (2020). The application of hyaluronic acid in bone regeneration. Int J Biol Macromol.

[30] Anderson HC (2003). Matrix vesicles and calcification. Curr Rheumatol Rep.

[31] (2016). Food and Drug Administration. Hylenex recombinant (hyaluronidase human injection) (FDA approved package insert).

[32] Shitsuka C, Palma LF, Pedron IG, Polotow TGG, Barros MP de, Leite MF (2020). Salivary profile of children with erosive tooth wear: a transversal study. Braz Oral Res.

